# Test-Retest Reliability of Home-Based Fitness Assessments Using a Mobile App (R Plus Health) in Healthy Adults: Prospective Quantitative Study

**DOI:** 10.2196/28040

**Published:** 2021-12-08

**Authors:** I-I Lin, You-Lin Chen, Li-Ling Chuang

**Affiliations:** 1 Recovery Plus Inc Chengdu China; 2 School of Physical Therapy & Graduate Institute of Rehabilitation Science, College of Medicine, Chang Gung University Taoyuan Taiwan; 3 Department of Physical Medicine and Rehabilitation, Chang Gung Memorial Hospital at Linkou Taoyuan Taiwan; 4 Healthy Aging Research Center, Chang Gung University Taoyuan Taiwan

**Keywords:** mobile health app, reliability, home-based fitness assessments, healthy adults, mobile phone, digital health

## Abstract

**Background:**

Poor physical fitness has a negative impact on overall health status. An increasing number of health-related mobile apps have emerged to reduce the burden of medical care and the inconvenience of long-distance travel. However, few studies have been conducted on home-based fitness tests using apps. Insufficient monitoring of physiological signals during fitness assessments have been noted. Therefore, we developed R Plus Health, a digital health app that incorporates all the components of a fitness assessment with concomitant physiological signal monitoring.

**Objective:**

The aim of this study is to investigate the test-retest reliability of home-based fitness assessments using the R Plus Health app in healthy adults.

**Methods:**

A total of 31 healthy young adults self-executed 2 fitness assessments using the R Plus Health app, with a 2- to 3-day interval between assessments. The fitness assessments included cardiorespiratory endurance, strength, flexibility, mobility, and balance tests. The intraclass correlation coefficient was computed as a measure of the relative reliability of the fitness assessments and determined their consistency. The SE of measurement, smallest real difference at a 90% CI, and Bland–Altman analyses were used to assess agreement, sensitivity to real change, and systematic bias detection, respectively.

**Results:**

The relative reliability of the fitness assessments using R Plus Health was moderate to good (intraclass correlation coefficient 0.8-0.99 for raw scores, 0.69-0.99 for converted scores). The SE of measurement and smallest real difference at a 90% CI were 1.44-6.91 and 3.36-16.11, respectively, in all fitness assessments. The 95% CI of the mean difference indicated no significant systematic error between the assessments for the strength and balance tests. The Bland–Altman analyses revealed no significant systematic bias between the assessments for all tests, with a few outliers. The Bland–Altman plots illustrated narrow limits of agreement for upper extremity strength, abdominal strength, and right leg stance tests, indicating good agreement between the 2 assessments.

**Conclusions:**

Home-based fitness assessments using the R Plus Health app were reliable and feasible in young, healthy adults. The results of the fitness assessments can offer a comprehensive understanding of general health status and help prescribe safe and suitable exercise training regimens. In future work, the app will be tested in different populations (eg, patients with chronic diseases or users with poor fitness), and the results will be compared with clinical test results.

**Trial Registration:**

Chinese Clinical Trial Registry ChiCTR2000030905; http://www.chictr.org.cn/showproj.aspx?proj=50229

## Introduction

### Background

Physical fitness plays an important role in overall health and quality of life and is directly related to physical activity [1]. Regular physical activity confers health benefits, such as increased life expectancy and reduced mortality [2,3]. However, the World Health Organization has reported that >80% of adolescents globally do not engage in sufficient physical activity. The prevalence of insufficient physical activity was 27.5% among adults aged >18 years worldwide [4]. Studies have indicated that physical inactivity is associated with poor physical fitness and increases not only the incidence and mortality rates of chronic disease, but also the medical and economic burden of disease [5-7]. Physical fitness has various degrees of influence on the activities and quality of life [1,8]. Poor physical fitness (below the 25th percentile of the fitness distribution) has a much greater impact on the risk of cardiovascular disease than insufficient physical activity [9]. Therefore, physical fitness needs to be considered as a fundamental assessment for people with a higher risk of chronic diseases.

Several physical fitness assessment methods have been established for reliability and validity. Physical fitness measures typically consist of cardiorespiratory fitness, muscle strength, endurance, agility, flexibility, and measures of body composition [1,10,11]. The 3-minute step test is one of the common cardiorespiratory fitness tests, consisting of stepping up and down a height of 23.0 cm-50.8 cm at a consistent step rate [12]. The 3-minute step test was shown to be reliable and valid in the general population and in patients with lung disease and rheumatoid arthritis [12-17]. Sufficient muscle power and endurance can reduce the risk of exercise injury and enhance cardiorespiratory capacity [18-20]. Wall squatting, push-up, and curl-up tests are common strength tests for the lower and upper extremities and abdominal muscles with established validity and reliability [11,21-23]. Balance and flexibility are important because poor stability may increase the risk of falls and limit functional activities [24-27]. Insufficient flexibility and mobility may restrict movement and cause pain [28-30]. Balance tests, the toe-touch test, the sit-and-reach test, and the Apley shoulder scratch test are common tests to assess balance, flexibility, and mobility [26,31-33]. However, most of these fitness tests are administered by a professional face to face, so patients or clients need to be present at a clinic or gym.

In consideration of cost and travel barriers, self-administered and home-based fitness tests may be more suitable for many people. However, there is currently little research on home-based fitness tests. One study showed that the home-based Senior Fitness Test, using inertia sensors and a depth camera, led to greater leg or arm strength, aerobic endurance, and flexibility [29]. The InterWalk Fitness Test incorporates indirect calorimetry and acceleration monitoring and was found to be accurate and reliable for persons with type 2 diabetes [34]. The self-administered Canadian Home Fitness Test was developed to assess cardiorespiratory endurance with a double 8-inch step and has an established record of safety and predictive ability [35-37]. Additional reliable home-based fitness tests that are easy to use and record data on accessible software platforms are needed.

As mobile technologies have advanced, an increasing number of health-related apps have emerged [38]. Some health apps provide patient education about lifestyle and health behaviors, some provide pain management, and others provide physical fitness assessments or interventions [34,38-40]. Among commercial fitness apps, most focus on cardiorespiratory fitness assessments, such as the submaximal walking data collected by a smartphone’s accelerometer [40]. Some apps focus on functional performance, such as movement speed or leg strength during functional activities [41]. However, most commercial fitness apps lack supporting evidence [40]. Only a few fitness apps have been tested for validity and reliability, and most are rated as having moderate to good validity [34,42-44]. Insufficient monitoring of physiological signals (eg, heart rate) during cardiorespiratory fitness assessment was noted among the available apps [40]. Therefore, we designed R Plus Health (Recovery Plus Inc), a digital health app that incorporates all the usual components of a fitness assessment but also monitors physiological signals.

### Objective

The aim of this study is to investigate the test-retest reliability of home-based fitness assessments using the R Plus Health app in healthy young adults.

## Methods

### Participants

A total of 31 participants were recruited with convenience sampling from 4 departments of a technology company in Chengdu, China. Sampling was performed via random draw. The inclusion criteria were healthy adults with normal health examinations, aged between 18 and 75 years, and with the ability to use smartphones. Those who rated more than 3 out of 10 on the visual analog pain scale; had poor compliance or were not willing to cooperate with the assessment; had regular strengthening sessions during the study period; had a history of alcohol abuse or illegal drug use; were pregnant, lactating, or trying to become pregnant; had participated in other clinical trials within 3 months before this study; and had uncontrolled chronic diseases were excluded. The participants received oral and written information about the study, and informed consent was obtained from all participants. This study was approved by the Chinese Ethics Committee of Registering Clinical Trials (ChiCTR2000030905).

### R Plus Health App

**Figure 1:**
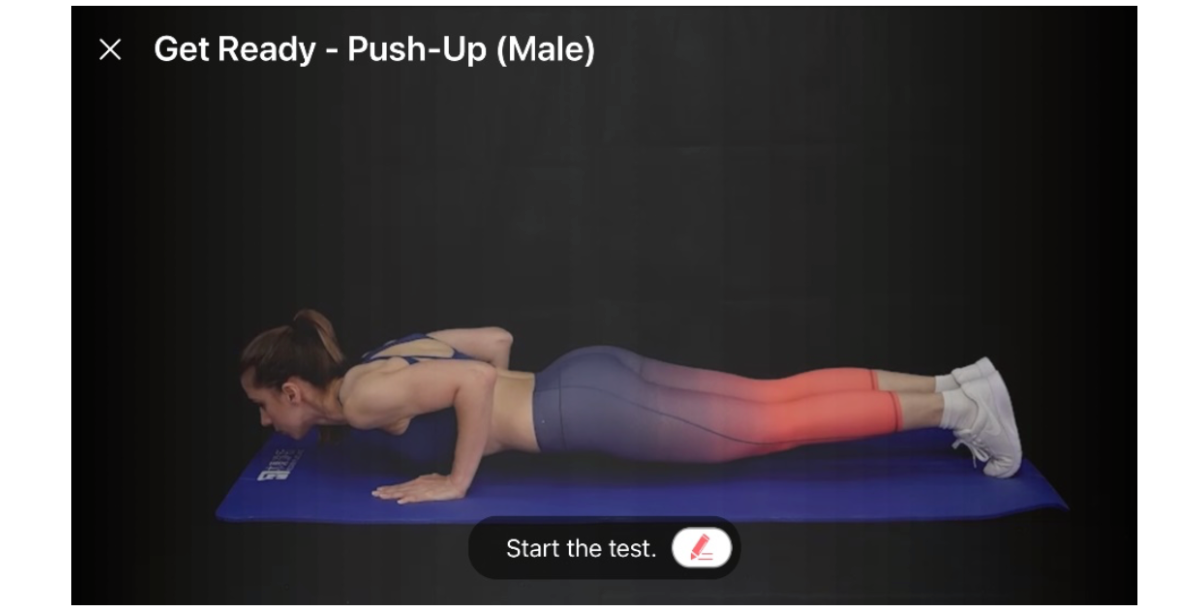
Video demonstration of the push-up test.

**Figure 2:**
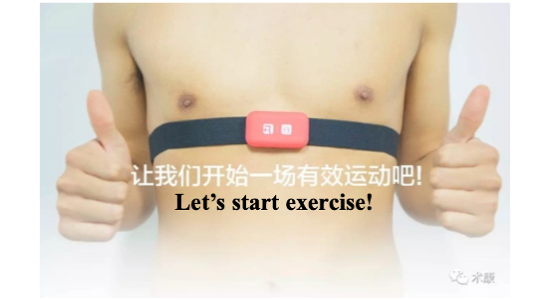
Demonstration of how to wear the heart rate monitor strap.

The R Plus Health app was developed as a tool for healthy adults and patients with chronic diseases. It includes fitness assessments and individualized exercise prescriptions with physiological signal monitors (eg, heart rate monitor). After downloading the R Plus Health app, the participants received an informed safety declaration and completed a health questionnaire, which was checked by doctors or other professional health care providers on the web. Through oral and video guidance, the participants were then instructed on how to perform the fitness assessments with maximal effort. The fitness assessments in the app included cardiorespiratory fitness, strength, balance, mobility, and flexibility tests (Figure 1). These fitness assessments have established clinical validity and reliability [12-25,30-33]. To complete the cardiorespiratory fitness test and record a real-time heart rate, the participants were required to wear a heart rate monitor below the sternum on a strap around the chest during testing (Figure 2). The heart rate monitor (Magene H64 dual protocol heart rate sensor) is compatible with the app and has Conformite Europeenne and Federal Communications Commission certification. Finally, according to the results of the fitness assessments and the overall health condition of each participant, a proper individualized exercise prescription was suggested by professional teams in the app.

### Assessment Procedures

Eligible participants enrolled in the study, provided informed consent, downloaded the R Plus Health app, and filled in the health questionnaire with the assistance of a physiotherapist. The physiotherapist recorded the basic data, including pain level on a visual analog scale and the overall health condition of the participants, at the baseline and final assessments.

All participants self-administered 2 fitness assessments with a 2- to 3-day interval between assessments to provide the best reproducibility [45]. The fitness assessments were administered sequentially (cardiorespiratory endurance, strength, flexibility, mobility, and balance). The participants followed the guidance and instructions in the app for each fitness assessment.

The 3-minute step test measures cardiorespiratory endurance based on how quickly the heart rate returns to normal after a 3-minute step exercise [12,13]. First, the heart rate monitor strap was worn for a 5-minute rest period beside the 30-cm step (to establish a baseline). After watching the tutorial videos in the app, the participants stepped up and down at 96 beats per minute (bpm) using a metronome for a total of 3 minutes. After finishing the test, the participants rested for 1 minute. The participants could suspend the test if any discomfort occurred.

The push-up and curl-up tests measure muscle strength and endurance in the upper limbs and abdomen, respectively, based on the number of completed repetitions [11,21,23,46]. When performing the push-up test, there were 2 variations in the starting position. The standard push-up test involved having the knees off the ground in the push-up position and was used for male participants. The modified push-up test involved having the knees on the ground and was used for female participants. The participants performed as many push-ups as possible with the correct form within 40 seconds. The curl-up test began with the participants lying on their back, knees bent at approximately 90°, feet flat on the floor, and arms straight with the palms of their hands resting on their thighs. The participants curled up and down at 40 bpm using a metronome. If the participants could not continue or stopped for more than 5 seconds, they clicked the *completed* button and recorded the repetitions.

The wall squatting test measures muscle strength and endurance in the lower limbs based on the holding time [11,22]. The wall squatting test began with the participants in a standing position, feet shoulder width apart and back against the wall; then, both knees were bent at a 90° angle. The participants held this squatting position for as long as possible. When the participants were finished, they could click the *completed* button and record the total time. If the participants held the position for more than 150 seconds, the app finished the test automatically.

The sit-and-reach test measures the flexibility of the hamstrings and the lower back with a ruler based on the distance [11,30,32]. The participants sat on the floor with their legs straight and their heels in line with a ruler, hands stacked, and palms facing downward. They then reached forward as far as possible along the measuring line. After reaching forward, the participants recorded their distance in centimeters.

The Apley scratch test or the upper extremity (UE) multipattern test measures the mobility of the upper limbs based on the distance between the middle fingers [33]. There were 2 patterns of upper limb flexibility: shoulder flexion, abduction, and external rotation and shoulder extension, adduction, and internal rotation. The participants performed these 2 patterns of movement for each upper limb and recorded the distance between both middle fingers. The results were classified as above average, normal, or below average.

The one-leg stance test measures balance based on the holding time [26]. The participants stood on one leg, bent the other leg 15-20 cm off the ground with their eyes open and their arms beside the hips. The participants maintained their balance for as long as possible. If the participants lost balance, they clicked the *completed* button, and the time was recorded in the app automatically. If the participants maintained balance for more than 30 seconds, the app finished the test automatically.

To minimize possible diurnal variation in physical fitness, the participants were instructed to perform the 2 assessments at the same time of the day. They were asked to avoid resistance training and exhausting work between assessments to minimize the potential effects of fatigue. After each test, the participants immediately recorded the results on paper to avoid recall effects and then sent them to the researchers. The researchers concealed the data of the participants in an envelope for anonymity and encoded the names as numbers to protect the privacy of the participants.

### Outcome Measures

At the baseline assessment, the descriptive data, pain score, and health condition of the participants were evaluated by a physiotherapist. Descriptive data included age, sex, height, and weight. The pain level was assessed using a visual analog scale from 0 (no pain) to 10 (worst pain). Health condition was assessed using a health-related questionnaire in the app and by a physiotherapist.

The outcomes of each fitness assessment included the raw data and converted score. The raw data were recorded as bpm, repetitions, seconds, and an ordinal scale. The converted scores (0-100) were computed using the app through normative data and a self-established score conversion system on the basis of the raw data.

The participants recorded the heart rate in bpm as raw data, and the converted scores used the same units. If someone could not complete the 3-minute step test, the reason was noted [12]. In each cardiorespiratory fitness test, 2 measurements were made: the average resting heart rate during the 5-minute rest period and the 1-minute recovery heart rate after the 3-minute step test.

The outcomes of the push-up, wall squatting, and curl-up tests were recorded as completed repetitions and total time. The flexibility of the lower limbs and lower back was measured in centimeters from negative to positive values. The mobility of the upper limbs was classified as above average, normal, or below average. The one-leg stance test recorded the total time in seconds [26,31-33].

### Data Analysis

Statistical analyses were conducted using SPSS 20.0 software (IBM Inc). Descriptive statistics were presented in the form of mean and SD, and the relative and absolute test-retest reliabilities of the fitness assessments were estimated separately.

#### Relative Reliability

The relative reliability of the fitness assessments was calculated using the intraclass correlation coefficient (ICC) with a 2-way mixed model (type absolute agreement). On the basis of the 95% CIs of the ICC estimates, agreement was rated as poor (<0.5), moderate (between 0.5 and 0.75), good (between 0.75 and 0.9), or excellent (>0.9) [47].

#### Absolute Reliability

The absolute reliability of the fitness assessments was evaluated using the SE of measurement (SEM), the smallest real difference (SRD), and Bland–Altman analyses [47,48]. The SEM expressed the measurement error variation between the assessments within a group and was calculated as SD_pooled_×√(1-ICC) [49]. In this formula, SD_pooled_ indicates the pooled SD for the 2 assessments. The SRD is a measure of sensitivity to change, represented as the magnitude of the change detected at a certain CI [50]. The SRD_90_ is defined as the SEM of the difference scores at a 90% confidence level and was calculated as 1.65×√2×SEM [48]. If the difference between the 2 assessments was greater than the SRD, it was interpreted as a real change. For all measurements, the smaller the SEM and SRD_90_, the greater the reliability.

The Bland–Altman analyses and plots assessed the agreement or repeatability of the 2 assessments [49,51]. They estimated the mean and SD difference between the 2 assessments and established limits of agreement (LOA) within a 95% CI [51]. The 95% LOA was calculated as the mean difference±(SD_diff_×1.96). The SD_diff_ indicates the SD of the difference between 2 measurements [48]. The scatter plots show the relationship between the difference between the 2 assessments (y-axis) and the mean of the 2 assessments (x-axis). More point scattering within the 95% LOA, along with a smaller range between the 2 limits, indicated a higher agreement [52,53].

## Results

### Overview

The characteristics of the participants and the descriptive statistics of the fitness assessments at baseline are shown in Tables 1 and 2, respectively. The study enrolled 31 participants (Table 1), which exceeded the minimum sample size of 26 (effect size of 0.5 and power of 0.8) calculated using G*power 3.1 [54]. The average age was 27.25 (4.0) years, and they had negligible pain (mean 0.19 out of 10 on the visual analog scale), which did not worsen during testing.

**Table 1:** Characteristics of the participants (N=31).

Characteristic			Values
Age (years), mean (SD)			27.25 (4.0)
**Sex, n (%)**			
	Female	16 (52)	
	Male	15 (48)	
Height (cm), mean (SD)			168.66 (7.61)
Weight (kg), mean (SD)			60.23 (11.41)
BMI (kg/m^2^), mean (SD)			21.03 (2.75)
Health status			Normal health examination
Pain assessment (range 0-10), mean (SD)			0.19 (0.65)

**Table 2:** Fitness assessments of the participants at baseline (N=31).

Domain and test items				Raw data, mean (SD)		Converted score^a^, mean (SD)	
**Cardiovascular fitness**							
	HR^b^ at rest^c^ (bpm^d^)			74.81 (9.6)		51.23 (16.5)	
	1-minute HR after test^e^ (bpm)			92.26 (18.3)		60.19 (19.1)	
	UE^f^ strength: push-up (repetitions)			12.94 (9.3)		58.71 (18.3)	
	Abdominal strength: curl-up (repetitions)			19.55 (13.7)		51.29 (18.8)	
	LE^g^ strength: wall squatting (seconds)			63.03 (26.1)		53.23 (18.1)	
	LE flexibility: sit-and-reach (centimeters)			2.85 (14.2)		57.74 (25.3)	
**Balance ability**							93.23 (19.4)
	Right leg stance (seconds)			31.77 (14.4)		—^h^	
	Left leg stance (seconds)			30.55 (9.9)		—	
**UE mobility^i^**							65.48 (18.8)
	**UE multipattern (above average), n (%)**					—	
		Right UE	23 (74)				
		Left UE	16 (52)				
	**UE multipattern (normal), n (%)**					—	
		Right UE	3 (10)				
		Left UE	6 (19)				
	**UE multipattern (below average), n (%)**					—	
		Right UE	5 (16)				
		Left UE	9 (29)				

^a^Converted score (0-100) from raw data in the app using normative data.

^b^HR: heart rate.

^c^Resting heart rate measurement.

^d^bpm: beats per minute.

^e^Heart rate recovery 1 minute after the 3-minute step test.

^f^UE: upper extremity.

^g^LE: lower extremity.

^h^Not available; no converted score was calculated respectively because the scores were averaged in balance ability.

^i^The upper extremity multipattern test was categorized into 3 classes (above average, normal, and below average).

Table 2 shows the results of the baseline fitness assessments as raw data (mean [SD]) and converted score (0-100). At the baseline assessments, the average heart rate was 74.81 bpm at rest, and the 1-minute recovery heart rate was 92.26 bpm after the 3-minute step test. In the strength tests, the average number of completed repetitions was 12.94 push-ups and 19.55 curl-ups, and the average holding time for the squatting test was 63.03 seconds.

### Relative Reliability

Table 3 summarizes the test-retest reliability of all the fitness assessments. On the basis of the raw data, the ICCs for all tests were 0.8-0.99. On the basis of the converted scores, the ICCs for all tests were 0.69 to 0.99. In most tests, the 95% CI was >0.5.

**Table 3:** Test-retest reliability of the fitness assessments (N=31).

Test items	ICC^a^ for the raw data	ICC for the converted score
HR^b^ at rest^c^	0.80 (0.58-0.90)	0.69 (0.34-0.85)
1-minute HR after test^d^	0.92 (0.84-0.96)	0.82 (0.63-0.92)
UE strength^e^	0.97 (0.94-0.99)	0.97 (0.93-0.99)
Abdominal strength^f^	0.98 (0.95-0.99)	0.94 (0.87-0.97)
LE strength^g^	0.93 (0.85-0.96)	0.82 (0.63-0.92)
LE flexibility^h^	0.89 (0.77-0.95)	1
UE mobility^i^	N/A^j^	0.99 (0.98-0.99)
Right leg stance	0.99 (0.98-0.99)	0.75 (0.5-0.88)
Left leg stance	0.89 (0.77-0.95)	0.75 (0.5-0.88)

^a^ICC: intraclass correlation coefficient (at a 95% CI).

^b^HR: heart rate.

^c^Resting heart rate measurement.

^d^1-minute HR after test: heart rate recovery 1 minute after the 3-minute step test.

^e^UE strength: upper extremity strength (push-up test).

^f^Curl-up test.

^g^LE strength: lower extremity strength (wall squatting test).

^h^LE flexibility: lower extremity flexibility (sit-and-reach test).

^i^UE mobility: upper extremity mobility (upper extremity multipattern test).

^j^N/A: not applicable; no intraclass correlation coefficient value was calculated because the raw data of the upper extremity mobility test was the percentage of participants, not a continuous variable.

### Absolute Reliability

The absolute reliability and Bland–Altman analyses are presented in Table 4. The SEM and SRD_90_ were 1.44-6.91 and 3.36-16.11, respectively, across the different assessments. The mean differences in UE strength, lower extremity (LE) flexibility, and right leg balance tests were close to 0. The 95% CI of the mean difference contained 0, indicating no significant systematic error between the 2 assessments in strength (−6.28 to 3.89 in the LE strength test and −1.54 to 0.89 in the UE strength test), flexibility (−2.65 to 3.57 in the LE flexibility test), and balance tests (−1.75 to 0.07 in the right leg stance test and −5.58 to 0.93 in the left leg stance test).

**Table 4:** Absolute reliability of the fitness assessments in raw data.

Raw data of test items	SEM^a^	SRD_90_^b^	Bland–Altman analyses					
			d^c^	SD_diff_^d^	SE of d^e^	95% CI of d	LOA^f^
HR at rest^g^ (bpm^h^)	4.28	9.99	5.61	5.58	1.00	3.57 to 7.66	−5.32 to 16.55	
1-minute HR after test^i^ (bpm)	5.18	12.08	7.19	6.91	1.24	4.66 to 9.73	−6.34 to 20.73	
UE strength^j^ (repetitions)	1.61	3.76	−0.32	3.31	0.59	−1.54 to 0.89	−6.81 to 6.17	
Abdominal strength^k^ (repetitions)	2.08	4.85	−1.74	4.00	0.72	−3.21 to −0.27	−9.58 to 6.10	
LE strength^l^ (s)	6.91	16.11	−1.19	13.87	2.49	−6.28 to 3.89	−28.38 to 25.99	
LE flexibility^m^ (cm)	4.71	10.99	0.46	8.47	1.52	−2.65 to 3.57	−16.14 to 17.06	
Right leg stance (s)	1.44	3.36	−0.84	2.48	0.45	−1.75 to 0.07	−5.70 to 4.02	
Left leg stance (s)	3.28	7.66	−2.32	8.87	1.59	−5.58 to 0.93	−19.70 to 15.06	

^a^SEM: SE of measurement.

^b^SRD_90_: smallest real difference at a 90% confidence level.

^c^d: mean difference between 2 trials.

^d^SD_diff_: SD of mean difference.

^e^SD_diff_/√n.

^f^LOA: limits of agreement (d±[SD_diff_×1.96]).

^g^HR at rest: resting heart rate measurement.

^h^bpm: beats per minute.

^i^1-minute HR after test: heart rate recovery in 1 minute after the 3-minute step test.

^j^UE strength: upper extremity strength (push-up test).

^k^Abdominal strength: curl-up test.

^l^LE strength: lower extremity strength (wall squatting test).

^m^LE flexibility: lower extremity flexibility (sit-and-reach test).

**Figure 3:**
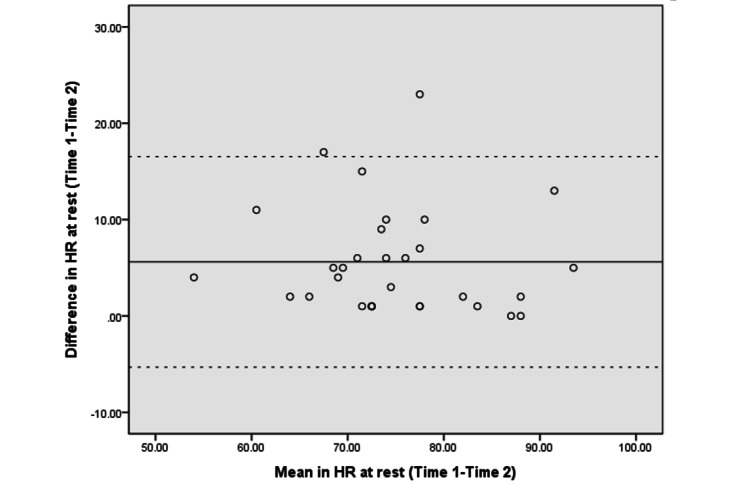
The Bland–Altman plots of differences between the 2 measurements in heart rate at rest. HR: heart rate.

**Figure 4:**
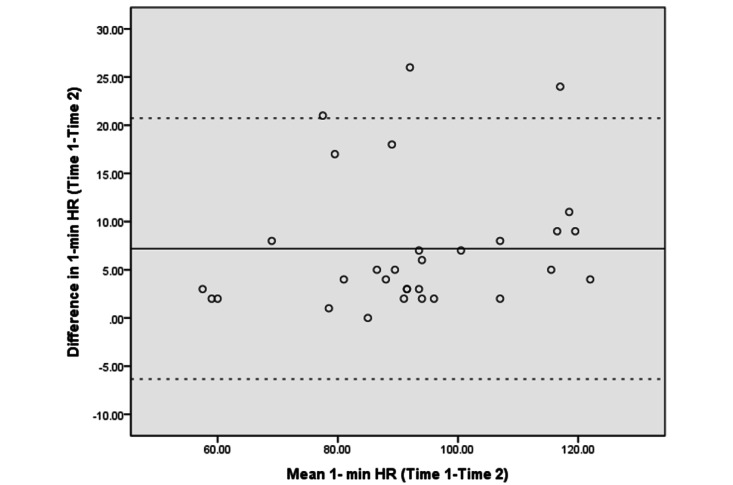
The Bland–Altman plots of differences between the 2 measurements in 1-minute heart rate recovery. HR: heart rate.

**Figure 5:**
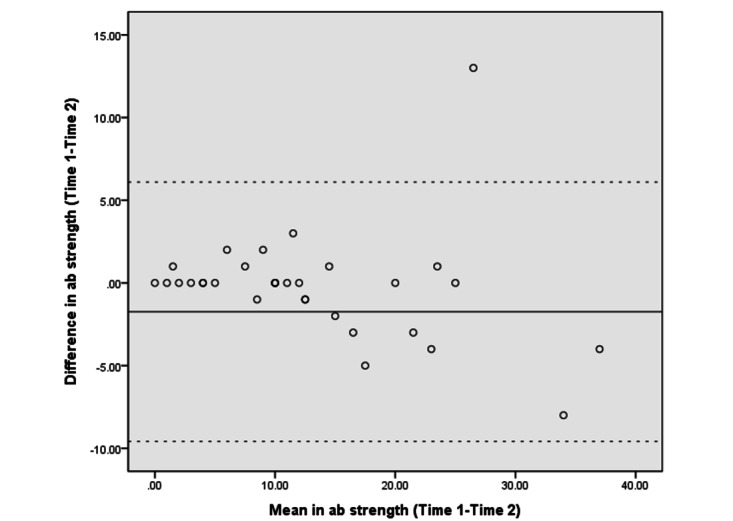
The Bland–Altman plots of differences between the 2 measurements in abdominal strength assessments. ab: abdominal.

**Figure 6:**
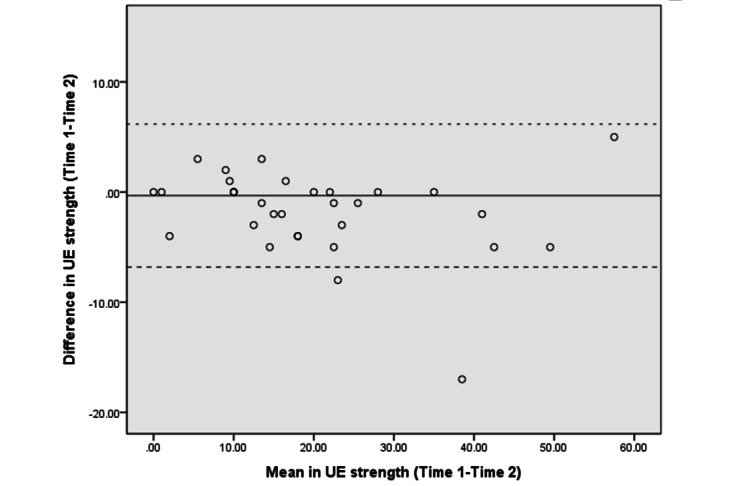
The Bland–Altman plots of differences between the 2 measurements in upper extremity strength assessments. UE: upper extremity.

**Figure 7:**
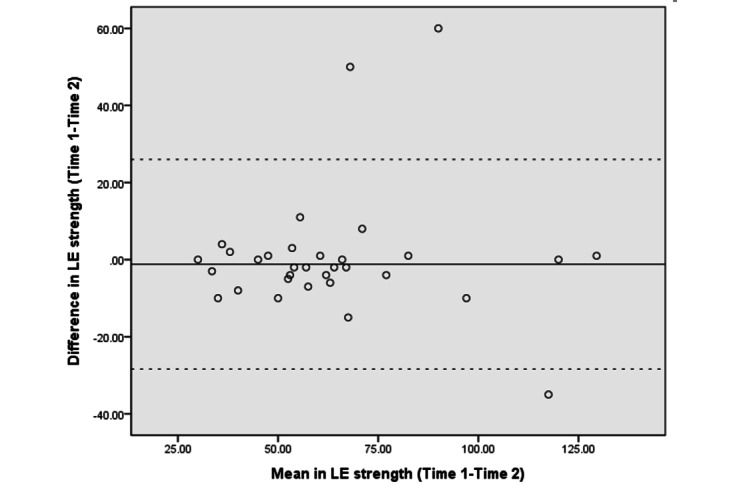
The Bland–Altman plots of differences between the 2 measurements in lower extremity strength assessments. LE: lower extremity.

**Figure 8:**
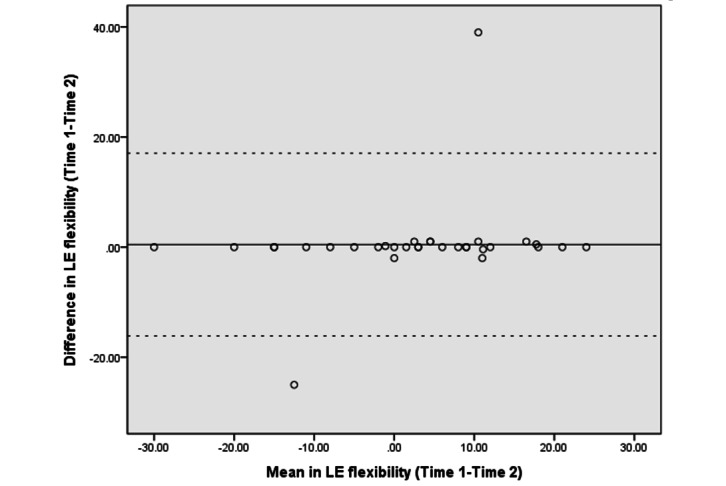
The Bland–Altman plots of differences between the 2 measurements in LE flexibility tests. LE: lower extremity.

**Figure 9:**
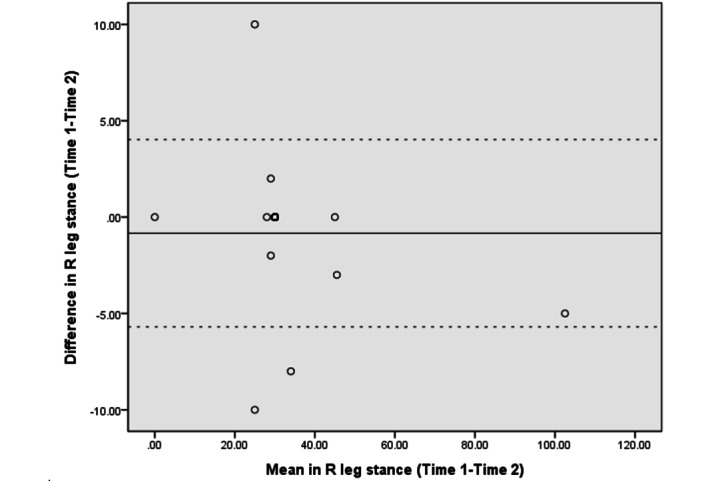
The Bland–Altman plots of differences between the 2 measurements in right leg stance tests. R: right.

**Figure 10:**
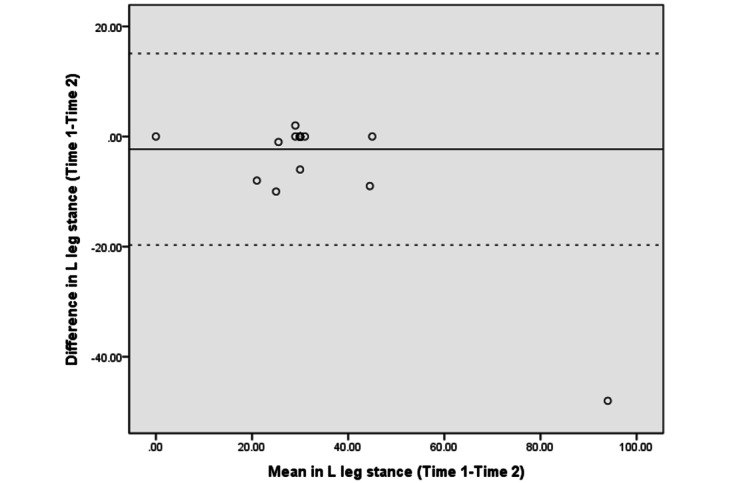
The Bland–Altman plots of differences between the 2 measurements in left leg stance tests. L: left.

Figures 3-10 show the Bland–Altman plots of the differences between the 2 measurements for all tests. Reference lines show mean differences between time 1 and time 2 (solid line) and 95% LOA for the mean difference (dotted lines). The differences for most of the tests were within the 95% CI. The LOA were −5.32 to 16.55 for the heart rate at rest and −6.34 to 20.73 for the 1-minute heart rate after test. The LOA were −6.81 to 6.17 in the UE strength test, −9.58 to 6.10 in the abdominal strength test, and −28.38 to 25.99 in the LE strength test. The LOA were −16.14 to 17.06 in the LE flexibility test, −5.70 to 4.02 in the right leg stance test, and −19.70 to 15.06 in the left leg stance test. There were at most 3 outliers in the 1-minute heart rate after, LE strength, and right leg stance tests.

## Discussion

### Principal Findings

This is the first study to investigate the test-retest reliability of home-based fitness assessments using a mobile health app in young, healthy adults. The results showed a moderate to good reliability of the fitness assessments. Therefore, through video and oral guidance, the app was shown to be reliable when applied to young users.

The self-administered fitness assessments in the app were feasible, with a low risk of injury. All participants completed the fitness assessments with the guidance of the R Plus Health app. Although some participants enrolled in the study had mild pain, they did not worsen after the fitness assessment. In other clinical research, it has been shown that mobile apps are able to conduct ecological momentary assessments, manage, and monitor patients with good adherence, detect symptoms, and evaluate the condition of a patient [55-57]. Therefore, well-designed mobile apps could offer a feasible means of self-assessment for clients and clinicians.

The results of our study were consistent with those of previous reliability studies [14]. Among the fitness assessments in the app, the test-retest reliabilities were moderate to good in this study. On the basis of the raw data, the ICCs for all tests were 0.8-0.99, indicating good to excellent reliability. On the basis of the converted scores, the ICCs for all tests were 0.68-0.99, indicating moderate to good reliability. The 95% CIs were above 0.5 in most tests. One previous study investigated the reliability of web-based versus supervised cardiovascular fitness assessments using the Young Men’s Christian Association 3-minute step test for college students [14]. The results of that study showed that there were no significant differences in the recovery heart rate between the 2 groups and that self-assessed cardiovascular fitness measurements were reliable [14]. Another study investigating the reliability of the Chester Step Test in patients with chronic obstructive pulmonary disease showed good reliability (ICC>0.8) [58]. In a previous analysis of strength fitness tests, reliability was established in adolescents, with ICCs of 0.7-0.9 in push-up, curl-up, and wall squatting tests [59]. For balance tests, the ICCs of single-leg stance tests were found to be >0.77 in young adults using a computerized balance platform [60]. These findings suggest that, regardless of the methods of assessing fitness capacity (eg, web-based and supervised assessments), the use of standard procedures and precise guidance under signal monitoring can ensure an accurate measure of actual performance. Self-administered fitness assessments in the R Plus Health app can be one of these efficient and reliable methods.

In addition to the relative reliability, the absolute reliability can demonstrate the agreement and sensitivity of the mean differences between the assessments. The SRD is a measure of sensitivity to change and represents the magnitude of the change at a certain confidence level [50]. If the difference between 2 assessments was larger than the SRD, it could be considered a real change, and the smaller the SEM and SRD_90_ of the difference, the greater the reliability. In this study, the SEM and SRD_90_ ranged from 1.44-6.91 and 3.36-16.11, respectively. For example, if the change was more than 16.11 in the wall squatting test, it was considered real at a 90% confidence level. In this study, the SRD_90_ in the wall squatting, push-up, and curl-up tests were 16.11, 3.76, and 4.85, respectively. These values were greater than the between-assessment changes reported in a previous study, which were 6.2, 2.6, and 0.1 for the wall squatting, push-up, and curl-up tests, respectively [59]. Different results might be because of different populations, ages, and experimental designs.

The Bland–Altman analyses and plots were generated to measure the repeatability of 2 measurement systems or of several trials using one method [49,51]. The scattering of data points within the 95% LOA and a smaller range between the 2 limits indicated higher agreement [52,53]. The 95% CI of the mean difference contained 0, indicating no significant systematic error between the 2 assessments for the strength, flexibility, and balance tests. The range of the LOA was slightly narrower in the UE strength (−6.81 to 6.17), abdominal muscle strength (−9.58 to 6.10), and right leg stance (−5.70 to 4.02) tests, indicating higher agreement. There was at least one outlier in each fitness assessment, and at most 3 outliers (in the 1-minute heart rate, LE strength, and right leg stance tests), which might be due to familiarization or fatigue in the second test.

Sufficient physical fitness is critical in daily life. It can decrease the risk of cardiovascular disease, pain, and injuries and improve the performance of life activities [9,18-20]. From the results of the cardiorespiratory fitness assessments in this study, the mean heart rate after 1-minute recovery from the step test was 92.26 bpm, indicating above average fitness base on the normative data [61]. In the LE strength wall squatting test, the mean holding time was 63.03 seconds, indicating an average fitness level [62]. In the LE flexibility sit-and-reach test, the mean distance was 2.85 cm, categorized as an average fitness level [11]. Even though the enrolled participants were generally in good health, they were below average in some of the fitness components. In the push-up test for UE strength and the curl-up test for abdominal muscle strength test, the mean number of repetitions was 12.94 and 19.55, respectively, which was below average based on the normative data and showed a need for improvement [63,64]. In the single-leg stance balance test, the mean holding time was 30 seconds (31.77 seconds for the right leg and 30.55 seconds for the left leg), indicating a below average fitness level [65]. Lack of muscle strength and balance can increase the risk of falls, pain, and injuries and limit daily life activities [24-27]. Therefore, comprehensive fitness assessments are essential.

Each participant differed in their performance in the physical fitness assessments according to variable self-conditions between the 2 assessments, and the results also differed from one participant to another. Even in healthy participants without chronic diseases, mild pain can lead to low strength in the extremities. Pain can inhibit muscle firing, and the lack of muscle contraction can decrease the stability of the joints and in turn, produce pain [66]. In other situations, insufficient muscle strength can lead to poor cardiorespiratory fitness. Evidence has shown that muscular fitness is related to cardiovascular prognosis and mortality [67]. As a result, according to the individual situation, it is important to detect weaknesses in the fitness profile and provide proper assessments and advice to clients. Through a comprehensive fitness assessment composed of cardiovascular endurance, strength, flexibility, and balance tests, the R Plus Health app can provide clinicians with a complete picture of the clients’ fitness. Clinicians can then choose to provide other detailed assessments on the web or at the clinic, which not only increases the efficiency of the evaluations but also decreases the medical and economic burden.

### Limitations and Future Studies

This study had several limitations. First, the level of difficulty in similar assessments differed from one participant to another, which may lead to ceiling or floor effects. According to the individual situation, automatic adjustment of the grade of assessments will be essential. Second, it is difficult to ascertain the accuracy of the performance assessments in the app without professional supervision. That is, the results of the app might not be identical to testing under professional supervision. In this study design, the results from different testing situations (with or without supervision) could not be compared. One solution to this problem would be to apply suitable monitoring equipment (eg, motion capture analysis and artificial intelligence techniques) to increase the precision of the assessments. However, this creates an additional technological burden. Cross-validation of the outcomes collected by the app versus professional staff will be the subject of future studies. Third, the study recruited young, healthy adults, so the results of the fitness assessments should not be generalized to other populations, such as older adults or patients with chronic diseases. Therefore, the fitness assessments in the app need to be conducted in other populations to compare the results between the app and clinical testing. Testing of the R Plus Health app in additional populations will be conducted in the future.

### Conclusions

Home-based fitness assessments using a mobile health app were reliable and feasible in young, healthy adults. The results showed moderate to good reliability, and the testing process caused negligible pain effects. This study highlighted an important contribution of mobile health apps to health care, that is, that healthy adults can self-administer fitness tests and thereby reduce overall costs. The results of mobile fitness assessments can offer a reliable understanding of a person’s health condition and help prescribe a safe and suitable exercise training regimen. Expansion of the use of this technology to different populations (eg, patients with chronic diseases or users with poor fitness) will offer widespread benefits to both patients and the health care system.

## Abbreviations

bpm: beats per minute
ICC: intraclass correlation coefficient
LE: lower extremity
LOA: limits of agreement
SEM: SE of measurement
UE: upper extremity
